# Changes in the reproductive function and developmental phenotypes in mice following intramuscular injection of an activin betaA-expressing plasmid

**DOI:** 10.1186/1477-7827-6-63

**Published:** 2008-12-16

**Authors:** Mi-Nyeu Kim, Moon Nyeo Park, Hoi Kyung Jung, Chunghee Cho, Kelly E Mayo, Byung-Nam Cho

**Affiliations:** 1 Department of Life Science/Research Center for Biopharmaceutical Lead Molecule, The Catholic University of Korea, Bucheon, 420-743, Korea; 2 Department of Life Science, Kwangju Institute of Science and Technology (K-JIST), Kwangju, Korea; 3 Department of Biochemistry, Molecular Biology and Cell Biology, Northwestern University, Evanston, Illinois 60208, USA

## Abstract

**Background:**

The TGF-beta family protein activin has numerous reported activities with some uncertainty in the reproductive axis and development. The precise roles of activin in in vivo system were investigated using a transient gain of function model.

**Methods:**

To this end, an expression plasmid, pCMV-rAct, with the activin betaA cDNA fused to the cytomegalovirus promoter, was introduced into muscle of the female adult mice by direct injection.

**Results:**

Activin betaA mRNA was detected in the muscle by RT-PCR and subsequent Southern blot analysis. Activin betaA was also detected, and western blot analysis revealed a relatively high level of serum activin with correspondingly increased FSH. In the pCMV-rAct-injected female mice, estrus stage within the estrous cycle was extended. Moreover, increased numbers of corpora lutea and a thickened granulosa cell layer with a small antrum in tertiary follicles within the ovary were observed. When injected female mice were mated with males of proven fertility, a subset of embryos died in utero, and most of those that survived exhibited increased body weight.

**Conclusion:**

Taken together, our data reveal that activin betaA can directly influence the estrous cycle, an integral part of the reproduction in female mice and activin betaA can also influence the embryo development as an endocrine fashion.

## Background

Activin and inhibin were first identified as gonadal protein hormones that regulate the synthesis and secretion of follicle stimulating hormone (FSH) in the pituitary gland which in turn controls the gonadal function [1,2]. They are members of the transforming growth factor-β superfamily of proteins [3]. Activin and inhibin are generated through the combinatorial assembly of an α subunit and two highly related β subunits, β_A _or β_B _to generate inhibin A (αβ_A_), inhibin B (αβ_B_), activin A (β_A_β_A_), activin B (β_B_β_B_), and activin AB (β_A_β_B_). Recently, activin β_C_, β_D_, β_E _chains [4-6], and partially characterized activin AC (β_A_: β_C_) and activin BC (β_B_: β_C_) proteins have been reported, although they are not expressed in the gonad [7]. Outside the gonad, activin β_A _was reported to be expressed in early pre- and postimplantation mouse embryos [8-10], and to be involved in the formation of mesoderm [11], and in secondary body axis formation in chick [12], zebrafish [13], and amphibians [14]. Activin β_A _is also expressed in the pituitary, placenta, bone marrow, brain, and spinal cord although precise functions of extragonadal activin are unclear [15].

In the reproductive axis, it has been recognized that activin potentially has an endocrine and paracrine (or autocrine) functions. The endocrine function of activin was inferred from the fact that correlation between high activin and high FSH in the mid cycle and luteo-follicular transition period was observed [16]. The paracrine function of activin was inferred from the fact that antibodies to activin B suppressed FSH secretion from cultured rat pituitary cells [17]. Another paracrine role of activin related to the reproduction is controversially reported in the ovary within which activin inhibited follicular development [18] whereas activin induced proliferation of the granulosa cells [19,20]. Relating with the pregnancy, activin has been reported to have effects on embryonic development. Activin A increased the rate of morula formation and the velocity of embryonic cleavage in mice [21]. And activin also influenced body axis formation in chick [12], zebrafish [13], and amphibian [14] during embryo development as explained above. Our comprehensive understanding of the activin function which is mainly based on the in vitro experiment, however, is still uncertain in the context of individual organism. Thus, we need to reinvestigate the actual functions in the in vivo system. Transgenic animal is a good model for this.

As to the actual functions of activin, previous studies have attempted in the intact organism through gene disruption or transgenic overexpression approaches. However, perinatal or early embryonic lethality is observed in these cases, so further studies for the activin functions are limited in adult organ [22,23]. Recent conditional knockout of activin β_A _which revealed that activin influenced ovarian growth and differentiation have extended the studies in specific organ of an early stage of adult mice [24]. These approaches, however, essentially do not permit the role of activin in the adult animal. In order to overcome this limitation and to investigate the actual function of activin in the adult, we adopted an alternative approach as described in our previous report [25]. Briefly, we transiently expressed activin, a secreted factor, in muscle under the control of the cytomegalovirus (CMV) promoter and assessed its impact on peripheral physiology at specific stage of female mice. The expression of foreign genes using CMV promoter is reported to persist for at least 22 weeks [26] which is thought to be enough period for hormone to exert its action. Our result revealed that activin β_A _colud directly influence the estrous cycle in female mice and the embryo development as an endocrine fashion.

## Methods

### Animals and experimental design

**Figure 1:**
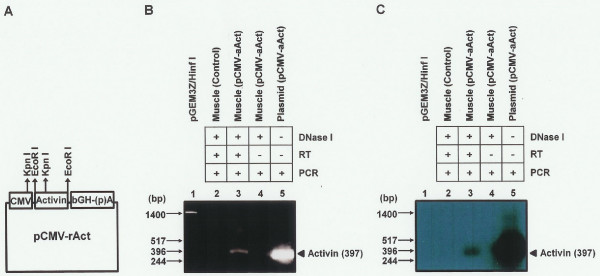
**pCMV-rAct structure and activin expression**. (A) Diagram of the pCMV-rAct construct. Functional elements include the cytomegalovirus (CMV) promoter, the activin cDNA, and the human growth hormone (hGH) poly(A) signal. (B) RT-PCR and (C) Southern blot analysis were performed as described in methods. Marker of pGEM3Z/infI was loaded in lane 1. RNA from the pCMV-rAct-injected mice was loaded in lane 3 after treatment with DNase I, reverse transcriptase, and PCR. RNAs from control mice and from the pCMV-rAct-injected mice without reverse transcription were used as a normal (lane 2) and an internal control (lane 4), respectively. pCMV-rAct plasmid was used as a positive control (lane 5). RT: reverse transcription, pCMV-rAct: pCMV-rAct-injected mice. +: treated (or reacted), -: not treated (or not reacted).

**Figure 2:**
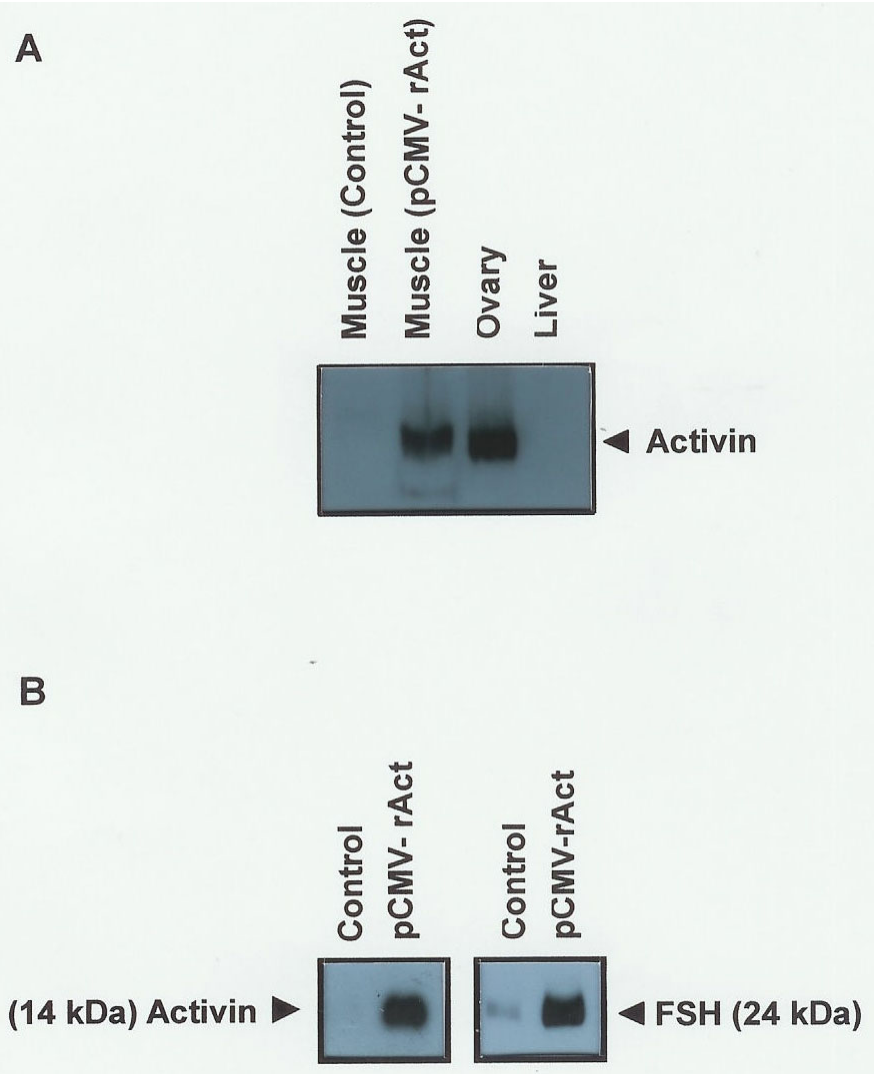
**Activin and FSH**. Western blot analysis was performed as described in methods. (A) Proteins from control muscle, ovary, and liver were used as a normal sample, a positive control, and a negative control, respectively. (B) One microliter of serum was obtained from the pCMV-rAct-injected mice, electrophoresed, and performed to western blot as described in methods. The western blot shown is representative of results obtained from three independent experiments.

**Figure 3:**
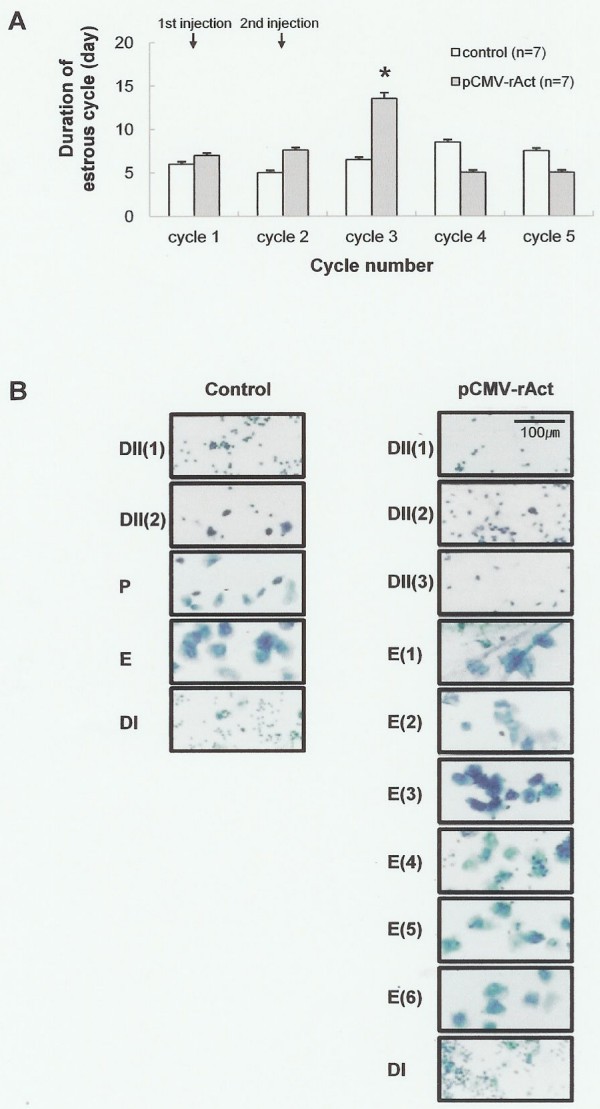
**Change in the estrous cycle**. (A) Each stage of the estrous cycle was identified by daily examination of vaginal cytology at 9:30 A.M. at a 100 × magnification. The first injection was carried out after confirming at least two normal estrous cycles and the second injection followed 4 days later. Note that the third estrous cycle was extended in the pCMV-rAct-injected female. The number of mice sacrificed is seven in each group (n = 7). Asterisks denote values that are significantly different from the mean control value (Student's *t*-test, *, *p *< 0.01). Values shown are mean ± standard deviation. (B) A photograph of one example. Of note is that extended estrus stage up to six days was observed. Control: vehicle-injected female mouse, pCMV-rAct: pCMV-rAct-injected female mouse. DI: diestrus I, DII(1): diestrus II day 1, DII(2): diestrus II day 2, P: proestrus, E: estrus, E(1–6): estrus day 1–6.

**Figure 4:**
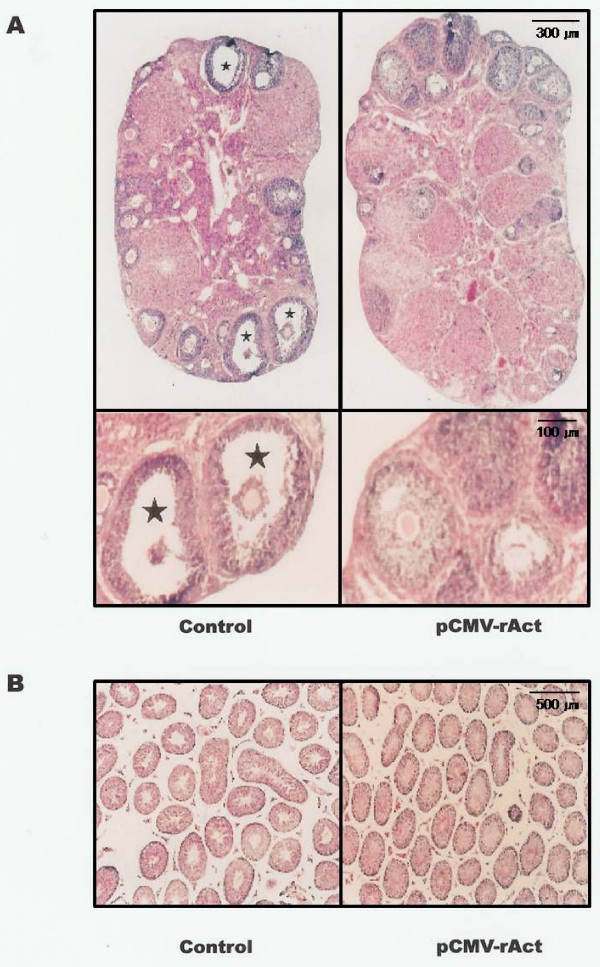
**Histology of the gonad**. The ovaries (A) and testis (B) were prepared as described in methods and observed at 40 × (A; Above, B) and 120 × (A; Below) magnification. Of note is that follicle revealed a thickened granulosa cell layer with a small antrum and increased numbers of corpora lutea were observed in the pCMV-rAct-injected female mouse. Asterisks indicate antra in the follicles of control mice.

**Figure 5:**
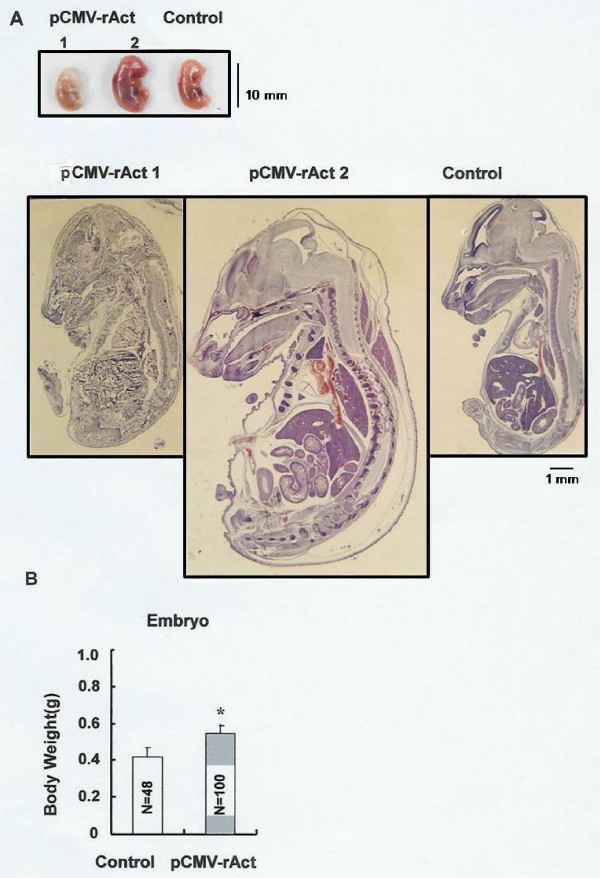
**Embryos from the pCMV-rAct-injected mice**. (A) The two surgically isolated embryos at left were obtained at E15.5 from a pCMV-rAct-injected female mouse, one of which was dead (pCMV-rAct 1) and the other of which was atypically large (pCMV-rAct 2). Tissues of the embryos were observed at 6× magnification under the stereomicroscope (Leica ME Apo). (B) The embryos were weighted at E15.5. The number of embryos from control and the pCMV-rAct-injected female mice is 48 (N = 48), and 100 (N = 100), respectively. Asterisks denote values that were significantly different from control mean values (Student's *t*-test, *, *p *< 0.01)

**Figure 6:**
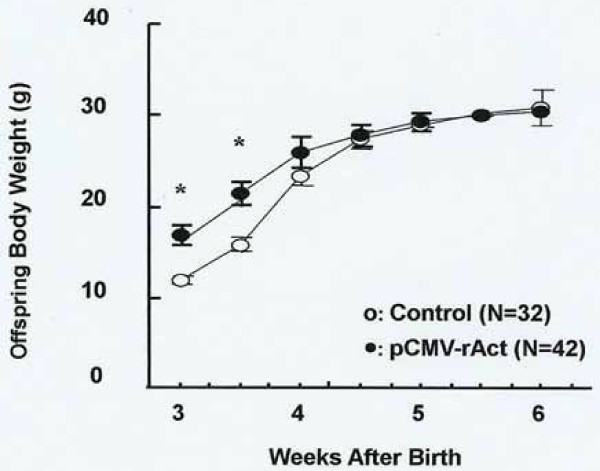
**Growth rates of offspring from the pCMV-rAct-injected mice**. Body weights of the offspring that were obtained from control and pCMV-rAct-injected mice are shown for the first 6 weeks after birth. The number of offspring used for control and pCMV-rAct-injected mice is 32 (N = 32) and 42 (N = 42), respectively. Asterisks denote values that are significantly different from control mean values (Student's *t*-test at each point, *, *p *< 0.01). Values shown are means ± standard deviation.

ICR mice at 2 months of age were purchased from the Daehan Animal Center and maintained under light under 14 h light, 10 h dark illumination at 23°C, with food and water available *ad libitum*. Plasmid DNA was purified and injected as described [25]. To measure activin β_A _mRNA and activin protein, a single injection of 300 μg pCMV-rAct in 50 μl of 10% sucrose in saline was performed at 10:00 A.M. and tissues were harvested 4 days after injection (Figs. 1B, 2A). For the detection of activin and FSH in serum, a single injection of 300 μg pCMV-rAct in 50 μl of 10% sucrose in saline was performed at 10:00 A.M. on diestrus II after confirmation of the two consecutive normal estrous cycles and serum was harvested 4 days later that is the diestrus II of the next cycle which normally reveals lower level of FSH in mice. Estrous cycle stages were determined by daily examination of vaginal cytology at 9:30 A.M. (Fig. 2B). For the reproductive studies, the first injection of 300 μg pCMV-rAct was performed at 10:00 A.M. on diestrus II and the second followed 4 days later. After the first injection of plasmid, estrous cycle stages were determined at 9:30 A.M. until next fifth cycle (Fig. 3). For the ovarian histology, female mice at diestrus I of the third cycle after injection of plasmid were sacrificed. For the testicular histology, the first injection was done and followed the second 4 days later. Then the mice were sacrificed 7 days later after the second (Fig. 4). For the developmental studies, the first injection was performed 1 day before mating, and the second 6 days after pregnancy was confirmed. Control mice were injected with the same amount of pcDNA3 vector (Figs. 5, 6). All the experiments were performed at least four times if not otherwise noted, and representative results are shown.

### Construction of the pCMV-rAct DNA expression vector

For the pCMV-rAct (6.9 kb), a 1.5-kb rat activin cDNA digested with EcoR I was cloned into the EcoR I site of a vector pcDNA3 (Invitrogen, USA) which contains a CMV early promoter and a bovine growth hormone polyadenylation site (Fig. 1A). The correct insertion of activin β_A _cDNA was confirmed by digestion with Kpn I.

### Reverse transcription-polymerase chain reaction (RT-PCR) and Southern blot hybridization

RT-PCR and Southern blot hybridization were performed as described [27]. Briefly, muscles were homogenized with denaturing solution (4 M guanidinium thiocyanate, 25 mM sodium citrate (pH 7), 0.5% N-lauryl sarcosine, and 0.1 M 2-mercaptoethanol). The homogenate was phenol/chloroform extracted, and the RNA precipitated and quantified with a U.V. 2000 spectrophotometer (Pharmacia, USA). A260/A280 ranged from 1.8 to 2.0. Ten micrograms of total RNA were used in duplicate. The RNA was then treated with DNase I (5 U, Promega, USA) at 37°C for 10 min in order to remove genomic and plasmid DNA, and reverse transcribed at 42°C with random hexamer primers and AMV reverse transcriptase (Promega, USA) in a 20 μl reaction. A mixture of oligonucleotide primers (500 ng each), dNTP, and Taq DNA polymerase (2.5 U) was added to each reaction, the total volume was brought to 100 μl with 1× PCR buffer [10 mM Tris (pH 8.3), 50 mM KCl, 1.5 mM MgCl_2_, and 0.01% gelatin] and the sample was overlaid with light mineral oil. Amplification was performed for 30 cycles using an annealing temperature of 65°C on an Omn-E thermal cycler (Hybaid Limited, UK). For the activin β_A _gene, the primers were designed to generate a 397 bp fragment. The 5' primer was 5'CCACACGACTTTTGCTGCCAG3' and the 3' primer was 5'GGTGATGATCTCCGAGGTCTG3'. After amplification, the samples were chloroform extracted, dried, resuspended in 10 μl TE buffer (10 mM Tris, pH 8.0, 1 mM EDTA), and electrophoresed on a 1.2% agarose gel. The gel was photographed after ethidium bromide staining. The PCR products were then denatured with sodium hydroxide and transferred to Nytran filters (0.45 μm, Schleicher & Schuell, Germany) under vacuum. They were hybridized with dixogenein-labeled activin β_A _cDNA, blotted with anti-dixogenein AP (1:1000) (Roche, Germany), washed, and exposed to X-ray film after blotting with CSPD (Roche, Germany).

### Protein blot analysis

Tissues were removed, homogenized in 400 μl of protein extraction buffer [0.1 M NaCl, 0.01 M Tris-Cl (pH 7.6), 1 mM EDTA (pH 8.0), 0.1% TritonX-100, 1 μg/ml aprotinin, and 100 ng/ml phenylmethylsulfonyl fluoride], and centrifuged four times. The homogenates were mixed with an equal volume of 2 × SDS-loading buffer [100 mM Tris-Cl (pH6.8), 200 mM DTT, 4% SDS, 0.2% BPB, 20% glycerol], placed in boiling water for 10 min, and centrifuged. The supernatants were transferred to fresh tubes. Samples of each extract containing 10 μg protein were heated at 70°C for 10 min, electrophoresed on a 12% acrylamide gel and transferred onto Nytran filters in transfer buffer (39 mM glycine, 48 mM Tris base, 0.037% SDS, 20% methanol). The blots were incubated overnight in blocking solution (5% nonfat dried milk, 0.02% sodium azide, 0.02% Tween) with shaking at 4°C, followed by exposure to primary activin β_A _antibodies (1:400) (Serotec, UK) overnight. They were washed in milk-TBS-Tween for 30 min and incubated with secondary anti-rabbit Ig horseradish peroxidase-linked whole donkey antibody (1:100)(Amersham Pharmacia Biotech, USA) in azide-free blocking solution [5% nonfat dried milk, 150 mM NaCl, 50 mM Tris-Cl (pH 7.5)] for 2 h. The secondary antibody-specific signal was detected with an ECL kit (Amersham Pharmacia Biotech, USA). For serum measurement of activin β_A _or FSH, one microliter of serum was obtained, electrophoresed, and western blot analysis was performed, using primary activin β_A _antibodies (Serotec, UK) or primary FSH antibodies (1:750) (Serotec, UK).

### Histology and statistical analysis

The gross appearance of embryos and excised tissues from injected and control mice were examined and the tissues immediately fixed in fresh 4% paraformaldehyde in PBS, pH 7.4. Following overnight fixation, they were dehydrated in ethanol and embedded in paraffin, and seven-micrometer sections were prepared with a microtome (Leica RM2235, Switzerland). The sections were de-paraffinized with xylene, dehydrated in absolute ethanol, and rehydrated in water. Sections were stained with hematoxylin, counterstained with eosin, and observed under a light microscope (Olympus IX70, Japan) or a steromicroscope (Leica ME Apo, Switzerland). For the statistical analysis, Student's *t *test was used for single comparison at α = 0.01. Statistics were performed no less than four independent experiments.

## Results

In initial studies, we tested whether intramuscular injection was an effective means of expressing activin β_A _in adult mice. At the transcription level, we investigated the expression of activin β_A_. After injection of the pCMV-rAct DNA construct (Fig. 1A), we obtained an expected 397 bp PCR product in mouse muscle using RT-PCR with activin β_A_-specific primers, indicating that it came from activin β_A _mRNA (Fig. 1B lane 3). Subsequently, we reconfirmed that the amplified PCR product really came from activin β_A _mRNA by Southern blot analysis with the labelled activin β_A _cDNA (Fig. 1C lane 3). At the protein level, western blot analysis revealed that mature activin β_A _protein (14 kDa) was synthesized specifically in muscle from pCMV-rAct-injected mice (Fig. 2A). Activin was also detected in the ovary, but not in the liver and control muscle. The level of serum activin β_A _protein was substantially elevated in the pCMV-rAct-injected mice (Fig. 2B left). Thus, both activin β_A _mRNA and protein were successfully expressed in muscle and secreted into the serum by this approach.

Activin was initially described and named for its ability to enhance FSH secretion in the female reproductive axis. To investigate whether the ectopically expressed activin β_A _influences serum FSH, we measured FSH (24 kDa) by western blot analysis and found an increase in serum FSH in the injected mice (Fig. 2B right). The induced activin β_A _(14 kDa) in serum was also found high at this time which corresponded to the increased FSH (Fig. 2B left). For the detection of FSH in serum, a single injection of pCMV-rAct was performed at 10:00 A.M. on diestrus II after confirmation of the two consecutive normal estrous cycles and serum was harvested 4 days later that is the diestrus II of the next cycle which normally reveals low level of FSH in mice. To examine the consequences of this altered FSH and activin levels, we examined the estrous cycle. The result was that the estrous cycle was extended at the third one after two injection of the plasmid (Fig. 3A). Within the estrous cycle, estrus stage was mainly influenced. One case is that estrus stage was extended up to 6 days and diestrus II was extended up to 3 days (Fig. 3B). The seemingly normal epithelial cells were clearly observed during 6 days.

When we observed the ovary, the size was slightly increased in the pCMV-rAct-injected mice. And the ovary in the pCMV-rAct-injected mice revealed a less cavity across the ovarian section compared to control which had small and numerous cavities. Moreover, increased numbers of corpora lutea were observed in the pCMV-rAct-injected mice, indirectly suggesting the increased ovulation (Fig. 4A, Above). Within the ovaries, the later follicular stages, including tertiary follicles, had a thickened granulosa cell layer with a small antrum. The oocytes appeared normal regardless of the abundant granulosa cells (Fig. 4A, Below). Activinβ_A _was expressed in the testis (15), implying some role. In order to gain some insight into the role of activin in male reproduction, we also applied our approach to the male mice. However, in case of male mice, any noticeable change in the histology was obtained except a slight increase in the number of seminiferous tubules of the testis in the pCMV-rAct-injected mice (Fig. 4B). We are under further investigation.

Since activin β_A _belongs to the TGF-β superfamily whose members are mainly involved in embryonic development [22], the possible role of activin β_A _in embryonic development was investigated. To do this, female mice were injected with pCMV-rAct while pregnant. The resultant offspring proved to be large; day E15.5 embryos were larger than similar aged control embryos (Fig. 5A pCMV-rAct2). Also offspring revealed a more accelerated development since more advanced vertebrae were observed. In addition, up to 10% of the embryos died (Fig. 5A). Histological analysis at embryological day 15.5 (E15.5) revealed severe tissue degradation across the body in the dying embryos (Fig. 5A pCMV-rAct1). Abortions were often observed within the uterus of the pCMV-rAct-injected pregnant mice. All the prematurely delivered embryos died immediately after delivery (data not shown). The mean body weight of the surviving embryos was about 1.5 times heavier than that of controls (Fig. 5B). These heavier body weights were maintained until 3 weeks after birth, but converged to control values by 6 weeks after birth (Fig. 6).

## Discussion

To further understand the detailed physiological function of activin β_A _in mammalian reproduction and development, we applied a "transient gain of function" mouse model as described in our previous research [25], using naked DNA injection as a gene transfer method. Direct injection of DNA into mouse muscle led to the appearance of activin β_A _mRNA and protein, and had a variety of consequences, including changes in estrous cycle, ovarian histology, embryo size, and survival. This approach provides a relatively simple means of examining the roles of the activin β_A _subunit in adult females and in development that could be extended to other endocrine genes.

Activin was first identified as gonadal protein to stimulate FSH in the pituitary gland [1,2]. When we induced activin β_A _through direct DNA injection, FSH in serum of pCMV-rAct-injected female was increased, suggesting that activin can act endocrine factor in addition to the already known paracrine factor. The resultant FSH enhancement also suggests change of the estrous cycle. When we examined the estrous cycle by vaginal smear method, the expected extended estrus stage was observed. In this case, it was presumed that increased FSH by ectopic activin expression induced follicle development in the ovary and subsequently elevated estrogen influenced the uterine tissue, resulting in extended estrus. This mechanism might be different from that of inhibin – induced estrus extension [28] in that an inhibin α-induced extension was occurred without increase of FSH.

With respect to the role of activin in reproductive tissues of the female mice, controversial results have been reported. Activin inhibited follicular development [18] whereas activin induced proliferation of the granulosa cells with FSH [19,20]. Our results support the granulose cell proliferation by activin overexpression. It remains, however, unclear whether activin acts solely or in the presence of FSH since FSH levels were significantly increased following activin β_A _overexpression in pCMV-rAct-injected mice (Fig. 2B). The increased numbers of corpora lutea in pCMV-rAct-injected mice was also observed in intraovarian activin β_A _knockout mice [24]. However the inducing mechanism of corpus luteum seemed to be different in that our approach adopted the addition of activin β_A _whereas an intraovarian activin β_A _knockout approach did the loss of activin β_A_. It was inferred that our addition of activin β_A _induced more corpora lutea with normal degeneracy whereas conditional deletion of activin β_A _induced normal corpora lutea with blocked degeneracy, both resulting in net increased corpora lutea in number.

In male mice, the reproductive system seemed not to be influenced by the induced expression of activin β_A_. Male fertility was not changed in pCMV-rAct-injected mice. In contrast, male sterility was observed in transgenic mice expressing activin β_A _[23]. Although the FSH was increased by the excess activin β_A _as it is in the female, the actual amount of FSH might be not enough to influence the testis, either because there is a lesser induction of activin β_A _in male compared to female mice with unexplainable reason (under investigation) or because the sensitivity to FSH between the ovary and the testis might be different. Minor changes in the testis could be directly influenced by activin β_A _since the binding site of the activin was observed within the testis and testis cell [29,30].

Gene disruption studies suggest an essential role of activin β_A _in embryonic development [22]. Our results indicate that overexpression of activin β_A _during development also has deleterious effects. In our experiments, some embryos from litters of the pCMV-rAct-injected female mice died during development, and the remaining embryos were observed to be larger than controls at birth. The increased size of the surviving embryos might be explained by their ability to more effectively compete for nutrients once a subset of the embryos die. Alternatively, the increased size of some embryos may limit the nutritional supply to others, contributing to the premature lethality that is observed. Collectively, these data indicate that activin β_A _is essential during development, but it is important that its levels be tightly regulated, and excessive amounts of activin are toxic or lethal. Consistent with the essential role of activin β_A _and tight regulation of activin β_A _expression, it was reported that both activin β_A _and activin β_B _are needed for development although activin β_A _at least is maintained over a minimum threshold level for normal growth and survival [31].

The larger size of the surviving mice in our study stands in contrast to the small-sized activin β_A _overexpressing transgenic mice observed at 3 weeks of age in another study [32]. In this case, the cause of the decreased size was presumed to be a thicker tongue and therefore a reduced ability to suckle. Another phenotype reported in activin β_A_-overexpressing mice [32], replacement of fatty tissue by connective tissue and a thickening of the epidermis, were not observed in pCMV-rAct-injected mice.

In addition to contributing new information on the consequences of altered activin β_A _expression in the adult female, these studies provide several important technical advances as previously reported [25]. Briefly, our approach is a very simple method and can be applied to many different species and to multiple strains of mice. For secreted proteins, our approach can be used effectively to study the phenotypic consequences of protein overexpression without the time-consuming production of transgenic mice. Moreover, the certain function of the protein in adult which was blocked by the deleterious effect during growth period in the overexpressing transgenic animal such as activin β_A_-transgenic animal can be overcome by this transient expression method.

## Conclusion

Our data revealed that activin betaA could directly influence the estrous cycle, an integral part of the reproduction in female mice and activin betaA also could influence the embryo development as an endocrine fashion. Moreover, our approach was proved to be efficient for functional analysis of the secreted proteins as a transient gain of function model.

## Competing interests

The authors declare that they have no competing interests.

## Authors' contributions

BNC designed the study and drafted the manuscript. MNK performed experiments related to the gene construction and subsequent gene expression. MNP participated in investigating the estrouc cycle. HKJ participated in investigating embryo. CC and KEM helped to draft the manuscript. All authors read and approved the final manuscript.
